# “Silent Echoes of the Day: Dream Content Analysis in Amyotrophic Lateral Sclerosis”

**DOI:** 10.1002/brb3.71387

**Published:** 2026-05-08

**Authors:** Alessandro Bombaci, Alessandra Giordano, Ilaria Lavalle, Alice Magnino, Andrea Calvo, Adriano Chiò, Alessandro Cicolin

**Affiliations:** ^1^ Neurology Unit IRCSS Policlinico San Donato San Donato Milanese Italy; ^2^ Vita‐Salute San Raffaele University Milan Italy; ^3^ Turin ALS Centre, “Rita Levi Montalcini” Department of Neuroscience University of Torino Turin Italy; ^4^ Sleep Medicine Centre, “Rita Levi Montalcini” Department of Neuroscience University of Torino Turin Italy

**Keywords:** ALS, dream, neurodegeneration, sleep

## Abstract

**Background:**

Amyotrophic Lateral Sclerosis (ALS) is a progressive neurodegenerative disorder characterized by the degeneration of upper and lower motor neurons, leading to muscle atrophy, weakness, and respiratory failure. Numerous studies evaluated the impact of diseases on dream content, and the dream content analysis may be considered an interesting tool in the study of the internalization of the consequences of significant life changes. The study of ALS patients’ dream content has been mostly neglected in the literature. This study investigated the dream content in a population affected by ALS.

**Material and Methods:**

We evaluated all consecutive outpatients referred to our ALS Centre using a weekly diary of dreams. Dream contents were coded according to the Hall and Van de Castle coding system.

**Results:**

Sixty‐eight patients completed the study. We collected 127 dreams (females 39.4%) (males 60.6%). Males showed a reduced presence of friends, anatomical elements, aggression, friendship, and sexuality. Instead, we found an increased presence of family members, situations in which the dreamer initiates aggressive action and familiar settings. In the female sample, we found a decreased presence of friends, aggressive and friendly elements, sex‐related content, and misfortune, while an increase in animal content.

**Conclusions:**

Our results demonstrate that dream content in ALS patients differs from that of healthy subjects, and we noticed some gender differences among ALS patients. The dream content can offer insights into ALS patients’ mental state and may improve clinicians’ ability to support their patients during their therapeutic course.

## Graphical abstract

ALS reshapes waking life through motor decline, social withdrawal, loneliness, and altered body image. Consistent with the continuity hypothesis, these changes may persist in dreams. In 68 ALS outpatients, a 7‐night dream diary yielded 127 reports, coded with the Hall–Van de Castle system (DreamSAT). Across genders, dreams showed fewer friends and reduced social interaction, with lower aggression/friendliness and diminished sexual content. Women dreamt more animals and less misfortune. Men showed more family members and aggressor roles, with fewer torso/anatomy references and familiar settings. Dream content may reveal patients’ emotional processing and guide tailored psychosocial care.

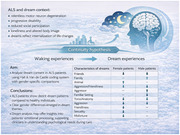

## 1 Introduction

ALS, often referred to as Lou Gehrig's disease, is a fatal neurodegenerative disorder affecting motor neurons, leading to the progressive degeneration of voluntary muscles, including respiratory muscles, eventually causing death (Hardiman et al. 2017). The disease has no known cure, and its etiology remains largely unclear, although genetic and environmental factors are involved. Respiratory insufficiency due to diaphragm weakness is a hallmark of ALS and significantly disrupts sleep architecture, with patients frequently experiencing sleep‐disordered breathing such as obstructive sleep apnea (OSA) or hypoventilation. These disruptions lead to fragmented sleep, nocturnal awakenings, and daytime sleepiness, contributing to a decreased quality of life.

The complex relationship between neurological diseases and sleep has been the subject of multiple studies, especially considering the role of Rapid Eye Movement (REM) sleep and its relation to emotional processing (Revonsuo 2000). Sleep is crucial for emotional regulation, memory consolidation, and cognitive function, which are significantly impacted in neurodegenerative diseases (Gnoni et al. 2023). Several neuroimaging studies have demonstrated that, during REM sleep, regions of the brain associated with emotional regulation—such as the amygdala and hippocampus—are active, suggesting that sleep plays a fundamental role in re‐evaluating and processing emotions experienced during wakefulness (Kussé et al. 2010). From this perspective, we can imagine dreaming as a “background noise” associated with all these processes. Disruptions in neuronal circuits could explain the prevalence of vivid and often emotionally charged dreams in patients suffering from neurodegenerative diseases.

There is no unified consensus on the definition of a dream. A dream can be described as a state of consciousness characterized by internally generated sensory, cognitive, and emotional experiences occurring during sleep. It is sometimes defined as a form of mental activity during sleep, which can include either isolated thoughts and images or more narrative experiences in the form of a well‐organized mental action that generates believable simulations of the real world and can be consciously recalled. Another definition attributed to dreaming, which emphasizes reports of dream content, is involuntary but organized mental action that generates credible simulations of the real world (Domhoff 2001). Dreams demonstrate that our brain, disconnected from the environment, can create an entire world of conscious experiences and dreams reflect the organization and functions of the brain. Indeed, just like during wake, in dreams, different sensory modalities are represented. There is also a coherence between the subject's cognitive and neuronal organization during wake and while dreaming.

As long as Freud's interpretive theory prevailed in the study of dreams, the relation between dreaming and waking conceptions was mediated by the censure of latent content, with the meaning accessible only through psychoanalytical interpretation.

More recent neurobiological perspectives demonstrate a consistently different point of view. According to Hobson's dream conceptualization, the meaning of dreams should be considered clearly “transparent” and not elaborated (Hobson and Pace‐Schott 2002).

Consistent with Hobson's view, Hall and Van de Castle (1966) developed a specific coding system to study dream content. They assumed that “the frequency with which a dream element appears, reveals the concerns and interests of the dreamer,” providing the opportunity “to link dream content with the waking thoughts and behavior of the dreamer” (Domhoff 1996). Domhoff hypothesized a continuity between dreams and waking life, meaning that “the concerns people express in their dreams are the concerns they have in waking life” (Domhoff 2004).

Many studies evaluated the dream content in healthy volunteers to find common features in people's dreams (Aamodt et al. 2021; Chellappa et al. 2009) and the Hall and Van de Castle method has also been applied to analyze patients’ dreams (Fantini et al. 2005; Newton 1970).

Numerous studies have evaluated the impact of diseases on dream content, with particular reference to cancer (DeCicco et al. 2010; Giordano et al. 2021; Goelitz 2002) and to cancer therapies (Giordano et al. 2012). Dream studies suggest that, also in neurodegenerative disease, the subconscious processing of illness is often mirrored in dream imagery. Vivid, distressing, or disease‐related dreams may reflect an individual's struggle with the physical and emotional realities of living with a terminal condition​ (Cicolin et al. 2020).

As a result of these considerations, we hypothesized that the complex emotional and practical experience related to a fatal disorder diagnosis might be reflected in the oneiric content. Following Domhoff's hypothesis about the continuity between dreams and waking life, the analysis of dream reports may be useful in evaluating the internalization of these feelings, emotions, and thoughts.

While existing literature supports the impact of neurodegenerative diseases on dream content, the specific effects in ALS patients remain underexplored and warrant further investigation. This study aimed to analyze the dream content in a cohort of ALS patients and compare the content to normative data on healthy subjects.

## 2 Materials and Methods

### 2.1 Design of the Study

Between July 2021 and December 2023, all consecutive outpatients referring to our regional ALS Expert Centre were evaluated. Inclusion criteria for the study consist of a probable or definite diagnosis of ALS according to the El Escorial criteria, disease duration of fewer than 36 months, non‐advanced clinical stage, and ability to understand and fulfill the study requirements and being able to personally give written informed consent, or through a legally authorized representative.

Disease severity was assessed using ALS Functional Rating Scale—revised (ALSFRSr) (Cedarbaum et al. 1999). The stage of disease was assessed using the KINGS system (Roche et al. 2012). Disease progression rate (PR) at the moment of dream diary filling was calculated as follows:

$$PR=\frac{48-\mathrm{ALSFRS}r}{\mathrm{Date}\;\mathrm{of}\;\mathrm{dream}\;\mathrm{diary}\;-\mathrm{Date}\;\mathrm{symptom}\;\mathrm{onset}}$$

ALS patients also underwent a neuropsychological evaluation (Iazzolino et al. 2019).

Exclusion criteria included: diagnosed sleep disorders, ongoing therapy with exogenous melatonin, and forced vital capacity (FVC) <60% of the expected value (indicates the maximum amount of air a person can expel from the lungs after deep breathing; it's an index of lung function).

Patients were asked to participate in the study during routine visits. Participation in the research was voluntary. The Local Ethics Committee approved the study (protocol number: 0028226, 2021), and all patients signed the informed consent form.

### 2.2 Data Collection

Each participant was provided with a structured dream diary, consisting of seven sheets, in which patients were asked to record all their dreams for seven consecutive nights, immediately after waking up in the morning at their home. Participants were encouraged to provide as much detail as possible, and family members or caregivers were allowed to assist in filling out the diary if necessary. We asked participants to return the completed diary during subsequent visits. In the event of non‐delivery, patients were contacted by telephone or e‐mail and asked to return the diary.

Because dream reports were nested within participants and the number of dreams per participant was uneven, results based on pooled dream counts may overweight individuals with higher recall frequency. We therefore report pooled HVdC/DreamSAT indicators as exploratory group‐level signals and recommend replication using participant‐level aggregation and/or multilevel modelling.

### 2.3 Statistical Analysis

The dreams were coded by a sleep medicine board‐certified psychologist using Hall and Van de Castle's method. Difficult or ambiguous issues were resolved in group discussions. The alphanumeric codes were uploaded into a DreamSAT spreadsheet (free web version, available at www.dreamresearch.net), which organized the codes into five macro categories (**Table** **1**) and provided frequencies for the total series of dream reports and percentage calculations.

**TABLE 1:** Categories on DreamSAT.

**Characters**
Male/female percent
Familiarity percent
Friends percent
Family percent
Dead and imaginary percent
Animal percent
**Social interaction percents**
Aggression/friendliness percent
Befriender percent
Aggressor percent
Physical aggression percent
**Settings**
Indoor setting percent
Familiar setting percent
**Self‐concept percents**
Self‐negativity percent
Bodily misfortunes percent
Negative emotions percent
Dreamer‐involved success percent
Torso/anatomy percent
**Dreams with at least one**
Aggression
Friendliness
Sexuality
Misfortune
Good fortune
Success
Failure
Striving

*Source*: Derived by Cicolin, A., M. Boffano, G. Beccuti, R. Piana, and A. Giordano. 2020. End‐of‐Life in Oncologic Patients’ Dream Content. *Brain Sciences* *10*, no. 8: 505.

Normative comparisons were performed against the Hall/Van de Castle normative database as implemented in DreamSAT (**Table** **2**). The HVdC norms are derived from systematically coded dream reports collected primarily from young adult college samples, and have been replicated across independent cohorts. Nevertheless, these norms represent historical and socio‐demographically specific reference data; therefore, our findings should be interpreted as deviations from a standardized baseline rather than as differences versus an age‐, culture‐, and emotionally matched contemporary control group.

**TABLE 2:** Normative data.

	Male norms (%)	Female norms (%)	Effect size	*p*
**Characters**				
Male/female percent	67	48	+0.39	0.000^b^
Familiarity percent	45	58	−0.26	0.000^b^
Friends percent	31	37	−0.12	0.004^b^
Family percent	12	19	−0.21	0.000^b^
Animal percent	6	4	+0.08	0.037^a^
**Social interaction percents**				
Aggression/friendliness percent	59	51	+0.15	0.014^a^
Befriender percent	50	47	+0.06	0.517
Aggressor percent	40	33	+0.14	0.129
Victimization percent	60	67	−0.14	0.129
Physical aggression percent	50	34	+0.33	0.000^b^
**Self‐concept percents**				
Self‐negativity percent	65	66	−0.02	0.617
Bodily misfortunes percent	29	35	−0.12	0.217
Negative emotions percent	80	80	+0.00	0.995
Dreamer‐involved success percent	51	42	+0.18	0.213
Torso/anatomy percent	31	20	+0.26	0.002^b^
**Other indicators**				
Physical activities percent	60	52	−0.38	0.000^b^
Indoor setting percent	48	61	−0.26	0.000^b^
Familiar setting percent	62	79	−0.38	0.000^b^
**Percentage of dreams with at least one**				
Aggression	47	44	+0.05	0.409
Friendliness	38	42	−0.08	0.197
Sexuality	12	4	+0.31	0.000^b^
Misfortune	36	33	+0.06	0.353
Good fortune	6	6	+0.02	0.787
Success	15	8	+0.24	0.000^b^
Failure	15	10	+0.17	0.007^b^
Striving	27	15	+0.31	0.000^b^

*Note*: The *p* values are based on the formula for the significance of differences between two proportions. The effect size derives from Cohen's *h*. The *h* statistic is determined by the following formula: *h* = cos^−1^(1–2P1)—cos^−1^(1–2P2). P1 and P2 are proportions between 0 and 1, and the cos^−1^ operation returns a value in radians.

^a^ Significant at the 0.05 level.

^b^ Significant at the 0.01 level.

*Source*: Derived by Domhoff, G. W. 2003. The Scientific Study of Dreams: Neural Networks, Cognitive Development, and Content Analysis (1st ed.). American Psychological Association.

DreamSAT makes it possible to analyze the frequency and nature of social interactions in dreams, allowing us to identify significant patterns of behavior and compare the dreamer's behavior with the above‐mentioned reference data (Domhoff and Schneider, https://dreams.ucsc.edu/).

Thematic analysis was utilized to identify patterns not captured by the Hall and Van de Castle's method coding framework. The value of qualitative dream analysis lies in its ability to uncover topics that may not fit into predefined objective categories (Braun and Clarke 2006). In this study, we followed Braun and Clarke's stages for conducting thematic analysis. This analysis consists of the production of initial codes from the data. The next phase involves sorting the different codes into potential themes and collating all the relevant coded data extracts within the identified themes, then, naming the themes and eventually, with a set of fully worked‐out themes, making the final analysis and writing the report.

## 3 Results

Ninety‐six patients (64.5 years ± DS) diagnosed with ALS were recruited from the Regional ALS Expert Center. The sample included 57 males and 39 females, reflecting the general gender distribution of ALS, which tends to affect males more frequently than females. Of the 96 eligible outpatients, 68 completed the study and returned the dream diary (71%). 27 patients completed the diary with at least one dream (40%): 10 females (37%) and 17 males (63%). Forty‐one patients returned the diary without reporting any dreams (60%). Comparing the 41 ALS patients that returned the diary empty and the 27 that had at least one dream, we observed no differences (*p* > 0.1; **Table** **3**) both in demographic (age and sex) and in clinical data (site of onset, presence of genetic mutations, ALSFRSr, PR, KINGS, and FVC at the moment of dream diary administration). Nine of the 68 ALS patients (13%) showed an FTD pattern. There were no differences in FTD distribution between ALS dreamers and non‐dreamers.

**TABLE 3:** Participant demographic and clinical information.

	ALS (68)	ALS dream (27)	ALS no dream (41)	*p*‐value (dream vs. not dream)
**Sex (M/F)**	42/26	15/12	26/15	0.52^a^
**Age at T0 ± SD (year)**	65.4 ± 9.0	63.7 ± 9.7	66.4 ± 8.4	0.42
**Site of onset (spinal / bulbar)**	49/19	18/9	29/8	0.29^a^
**Presence of a genetic mutation**	8	3	5	0.90^a^
**FTD cognitive profile**	9	4	5	0.79^a^
**ALSFRSr_tot at diagnosis ± SD (points)**	39.8 ± 4.7	39.7 ± 3.9	39.8 ± 5.1	0.64
**PR at diagnosis** **± SD (points lost/month)**	0.73 ± 0.75	0.84 ± 0.92	0.67 ± 0.60	0.52
**KINGS stage at diagnosis (0/1/2/3/4)**	0/15/30/23/0	0/8/9/10/0	0/6/22/13/0	0.18
**FVC ± SD (%)**	91.5 ± 18.9	93.6 ± 20.0	90.3 ± 18.0	0.49

^a^ *χ^2^ test*.

ALSFRSr_tot: ALS Functional Rating Scale—revised total score; PR: progression rate; FVC: forced vital capacity; FTD: frontal temporal dementia; SD: standard deviation; n/a: not assessed.

Demographic and clinical characteristics are reported in **Table** **3**.

We collected 127 dreams: 50 dreams were reported by females (39%) and 77 by males (61%).

Data on the dreams of female (**Table** **4**) and male (**Table** **5**) patients are presented below.

**TABLE 4:** Characteristics of female patients’ dreams and comparison with female normative data.

	Female patients	Female norms	*p*
Characters Friends % Animal %	23 12	37 4	0.022* 0.017*
Social interaction percent Aggression/friendliness %	24	51	0.018*
Dreams with at least one Aggression % Friendliness % Sexuality % Misfortune %	10 22 00 18	44 42 0.4 33	0.000** 0.003** 0.010* 0.016*

^*^ Significant at the 0.05 level.

**TABLE 5:** Characteristics of male patients’ dreams and comparison with male normative data.

	Male patients	Male norms	*p*
Characters Friends % Family %	14 23	31 12	0.000** 0.004**
Social interaction percents Aggression/friendliness % Aggressor %	26 86	59 40	0.000** 0.009**
Settings Familiar setting %	81	62	0.008**
Self‐concept percent Torso/anatomy %	0	31	0.009**
Dreams with at least one Aggression % Friendliness % Sexuality %	10 23 1	47 38 12	0.000** 0.008** 0.000**

^**^ Significant at the 0.05 level.

Compared to normative data, the male patients showed a statistically significant decrease in the presence of friends, elements concerning the torso and anatomy in general, aggression, friendship, sexuality, and the aggression/friendliness ratio. Instead, we found increased presence of family members, situations in which the dreamer initiates aggressive action and familiar settings.

In the thematic dream analysis, we observed that hospitals were mentioned only on five occasions (4%). Healthcare personnel appeared in four dreams (3%), specifically a doctor, a pharmacist, a group of nurses, and a veterinarian. A syringe was referenced once. Additionally, deceased relatives and acquaintances, including parents, a pharmacist, a brother‐in‐law, and a former employer, were dreamt of on seven occasions (6%).

In the female sample, we found a statistically significant decrease in the presence of friends, aggressive and friendly elements, sex‐related content, misfortune, and a reduction in the aggression/friendliness ratio; on the other hand, there is an increased presence of animals compared to the normative data.

### 3.1 Discussion and Conclusions

This study was undertaken to investigate and analyze the distinguishing characteristics of dreams in people living with ALS compared to a normative, healthy sample. The results presented offer a unique contribution to the literature, as this is, to the best of our knowledge, the first study to examine the dreams of ALS patients, providing new perspectives on the dream experiences of this population.

The findings from this study provide evidence that the dream content of ALS patients is deeply intertwined with their emotional and psychological experiences. By systematically analyzing the dreams of individuals living with this debilitating disease, we gain valuable insights that may not only reflect their current state of mind but also suggest the profound psychological impact of living with a relentless, lethal condition.

Although a substantial proportion of patients with ALS did not report dream experiences, analysis of demographic variables and clinical characteristics (including the presence of cognitive impairment) showed no significant differences between patients who reported dreaming and those who did not.

Dream content is inherently state‐dependent; however, HVdC indicators are designed to capture proportional patterns that are more stable at the aggregated level than single‐dream narratives. Accordingly, our findings should be interpreted as cohort‐level tendencies during the collection period.

Quantitative dream analysis has revealed specific characteristics in the dream content of the study sample. Both male and female participants reported dreams featuring fewer familiar people, friends, social interactions, and friendliness. Contextually, a noteworthy abolition of sexual dream content needs to be observed, most of all in males. These data could be due to the tendency toward social withdrawal, likely intensified by the progressive physical and emotional challenges of the disease (Petitte et al. 2015).

This interpretation is supported by the observed decrease in both aggression and friendliness within dreams, suggesting a diminished emotional intensity in the dream life of ALS patients, which may mirror the reduction in meaningful social interactions and the emotional depletion often accompanying ALS‐related cognitive decline. Petitte and colleagues demonstrated that the majority of individuals living with chronic illnesses experience some degree of loneliness (Petitte et al. 2015). Loneliness, defined as “perceived social isolation” (Barlow et al. 2015), refers to “negative emotions that arise from a discrepancy between the desired and the actual quality or quantity of an individual's social relationships” (Barlow et al. 2015). Although age itself is a risk factor, it has been discussed that chronic illnesses represent an additional threat, contributing to loneliness. In the lives of patients with chronic diseases, pain and fatigue may limit daily activities, generating feelings of frustration, loss of control, and loneliness (Özkan Tuncay et al. 2018). For ALS patients, the perceived divergence from the healthy population and distancing by peers due to symptoms can further deepen this sense of isolation (Consonni et al. 2024). Additionally, healthy friends may distance themselves in response to the patient's symptoms or due to their discomfort with the illness, further contributing to the patient's feelings of isolation.

Upon detailed analysis, it is evident that in the male sample, there was a reduction in references to anatomy. It is conceivable that the disease induces a form of disconnection from one's body, which no longer occupies a central role in the patients' dream experiences. In the case of a progressive and debilitating disease, such as ALS, these changes can be particularly profound. The data suggest that the progression of the disease and the gradual loss of motor abilities may play a crucial role in altering the mental and dream representation of the body (Giordano et al. 2012), leading to a diminished centrality of bodily experiences in the lives of these patients: the body is no longer an active protagonist in their daily lives, but rather an unresponsive element. Moreover, similar to findings reported by Giordano and colleagues (Giordano et al. 2012), which highlighted that women who have undergone mastectomy report fewer dreams with sexual content, these results show an absence of references to sexuality in both patient samples. As suggested by Mayer and Eisenberg, “As an individual develops and transitions through states of health, illness, and sometimes disability, the concept of the body and physical self‐esteem must adapt to these changes” (Mayer and Eisenberg 1988). The loss of self‐confidence and of acceptance of the body that is changing due to the disease could be another reason for abolishing sexual desire. It is also possible that the near‐complete absence of sexual elements may indicate that patients were unwilling to disclose the sexual details of their dreams, especially in cases where caregiver assistance was required to transcribe the dreams. Such dreams are intimate and sensitive, often perceived as embarrassing or socially unacceptable (Yu and Fu 2011), and this may have discouraged patients from revealing such private details. In addition, the ALS patients' perceptions of mortality, due to their relentless progression of the disease, may contribute to the reduction of attention to the body and to focus on other objectives and interests.

Another important finding from this study is the increased representation of family members and familiar settings in the dreams of male ALS patients. This phenomenon mirrors the heightened presence of family members during waking hours, as these individuals often play a pivotal role in the care and support of patients throughout the disease progression (Primomo et al. 1990). In the context of chronic illness, the family support system represents a fundamental resource that works in tandem with healthcare systems. The family acts as a caregiver, emotional support, and a vital part of the patient's social environment (Boise et al. 1996). In the case of ALS, where no cure exists, it is the family that often takes care of the patients (Giusiano, et al. 2121). In support of this, the male sample also showed an increase in familiar settings. Domhoff's continuity hypothesis allows us to interpret this finding, suggesting that the presence of family support in the daily lives of ALS patients is reflected in their dreams through the increased presence of family members, caregivers, and familiar places, highlighting the importance of this type of support in their daily experience. The lack of increased family presence in the dreams of female patients may be explained by data from Hall and Van de Castle, which indicates that women are already more likely to dream of family members than men. Given that family members are already more present in female dreams initially, it is less likely that an additional increase would be observed, even in chronic illness situations such as ALS. Furthermore, it is plausible to consider the differing caregiving roles between Italian women and men aged 60–80 years. As evidenced in previous studies, female caregivers are more likely to assume the role of primary caregiver and often report a greater burden, particularly in the physical and social aspects of caregiving (Biliunaite et al. 2022; Del Río‐Lozano et al. 2022). Males in our cohort seem to be more dependent on their spouses during this chronic illness than women. Providing care for ALS patients requires sustained, intensive support, involving significant physical effort and emotional strain. Given that women are frequently engaged in additional household or familial responsibilities, this added caregiving role may contribute to overload and a reduction in social participation (Tramonti et al. 2015).

Through the thematic analysis and in contrast to what is observed in dreams of patients affected by oncologic conditions (DeCicco et al. 2010), in our cohort of ALS patients, we did not observe a tendency of dream healthcare professionals or hospital settings.

A recurrent topic within our sample involves the presence of deceased individuals in patients' dreams. Several participants frequently reported dreams in which deceased relatives, such as parents or other acquaintances, are alive and interact with them in meaningful ways. This fact could be comparable to the findings by Coolidge and Fish (1984), who documented that terminally ill patients often experience dreams where they are “visited” by deceased loved ones. In these dreams, deceased relatives offer companionship and support, symbolically accompanying patients as their illness progresses. Additionally, the presence of death within these dreams may reflect patients' perceptions of mortality.

This study reveals that male ALS patients demonstrate a higher frequency of aggressive interactions in their dreams. Aggression, widely recognized as the most prevalent form of social interaction in dreams (Hall and Van de Castle 1966), is particularly notable here. Conduit et al. (2000) suggest that negative experiences, being more salient both in wakefulness and dreams, are more readily recalled. For ALS patients, emotions such as anger, fear, and frustration may be amplified and reflected in dreams as aggressive acts, potentially as coping mechanisms. Anxiety and fear, heightened by the challenges of an ALS diagnosis, may contribute to this kind of behavior during dreaming; aggression often becomes a response when individuals face stressful, emotionally restrictive circumstances (Kashani et al. 1991). Chronic illnesses may further weaken emotional regulation, potentially leading to disorganized emotions and limited control, as Charles (2010) noted, which could exacerbate aggressive dream content. Furthermore, previous research indicates that aggressive dream content generally decreases with age, though this reduction is slower in men (Hall and Domhoff 1963). Men's aggressive dream content peaks between ages 2 and 12, declining gradually after 30. The slower decline in males compared to females, who typically exhibit lower levels of dream aggression, could explain why female participants demonstrated less aggressive content in their dreams.

The female sample reported a higher number of animals in their dreams. Some studies (Revonsuo 2000; Schredl 2013) highlight that animals tend to be more prevalent in children's dreams. From a Jungian perspective, this may represent a connection with the more primitive part of the mind, a form of regression understood as a return to a more primitive stage of functioning in response to significant stress—a topic widely discussed in the literature as a possible consequence of illness (Corradi 1983). According to the continuity hypothesis (Corradi 1983), dream content reflects waking life experiences, which may imply that the women in this study—possibly spending more time at home—were frequently exposed to animals. Schredl found that living with pets correlates with their increased presence in dreams, indicating that familiarity with animals could contribute to their dream prevalence (Schredl 2013).

This study demonstrates several significant strengths, notably its position as the first investigation into the dreaming patterns of individuals with Amyotrophic Lateral Sclerosis (ALS), coupled with a rigorous analytical methodology. However, it also presents certain limitations, including (A) a cross‐sectional design, (B) a single‐center framework, (C) a limited number of participants, and (D) the reliance on historical Hall/Van de Castle norms rather than a contemporaneous control group matched for age, culture, and psychosocial stressors.

Consequently, further research is essential, particularly longitudinal studies tracking changes in dream content as ALS progresses. Comparative studies with other neurodegenerative diseases could clarify whether these topics are unique to ALS or widespread across conditions. Exploring the dreams of caregivers could also provide valuable insights into the broader emotional landscape surrounding ALS. Understanding the psychological impact of caregiving, along with the dreams of both patients and caregivers, may uncover important dynamics that affect the overall quality of life for families dealing with this challenging condition.

Integrating dream analysis into clinical practice, alongside interdisciplinary approaches combining psychology, neurology, and sleep medicine, may support innovative therapies focused on enhancing emotional resilience and improving the quality of life for ALS patients and their families. Dream content analysis can reveal expressions of an inner sense of self through a novel analytical approach that can significantly enhance the clinician's ability to support patients throughout the treatment process. Indeed, providing appropriate psychological support is essential for patients who face significant challenges in their self‐perception and who may experience significant changes in their quality of life.

In summary, this study is the first to systematically examine the dream content of ALS patients, offering new insights into their emotional and psychological experiences. The findings suggest that the dreams of ALS patients are marked by a high prevalence of negative emotions related to a low presence of friends and friendliness, symbolic imagery related to the disease's progression, and a sense of perceived loneliness. The frequent presence of family members and the clear reduction of social interactions and sexual content suggest that ALS patients may feel emotionally isolated.

The gender differences observed in the dreams of ALS patients highlight the importance of tailoring psychological support to the individual needs of each patient. Male patients may benefit from interventions that address feelings of aggression and conflict, while female patients may require more emotional support and nurturing.

Future research should explore the longitudinal changes in dream content as ALS progresses, as well as the potential impact of different treatments, such as non‐invasive ventilation and psychological therapy, on dream content. Dream analysis could become a valuable tool in understanding the emotional experiences of ALS patients and improving the quality of care provided to them.

In conclusion, this study makes a novel contribution to the literature on dreams and the emotional experiences of ALS patients, suggesting that dream analysis could be integrated as a clinical tool for both patients and their caregivers.

## Author Contributions

Alessandro Bombaci: Conceptualization (lead), supervision (lead), data curation (equal), writing – original draft (lead), formal analysis (equal), writing – review and editing (equal), software (equal), methodology (lead). Alessandra Giordano: Conceptualization (equal), data curation (lead), writing – original draft (equal), formal analysis (lead), review and editing (equal), resources (equal), methodology (equal). Ilaria Lavalle: Data curation (equal), formal analysis (equal), writing – original draft (equal), review and editing (equal), resources (lead). Alice Magnino: Data curation (equal), review and editing (equal). Andrea Calvo: Review and editing (equal). Giuseppe Fuda: Review and editing (equal). Andrea Calvo: Supervision (equal), writing – review and editing (equal). Adriano Chiò: Supervision (equal), project administration (equal), writing – review and editing (equal). Alessandro Cicolin: Supervision (equal), project administration (lead), writing – review and editing (equal).

## Conflicts of Interest

The authors declare no conflict of interest.

## Funding

The authors have nothing to report.

## Informed Consent Statement

Ethical approval was granted by the Turin ALS Centre's Ethics Committee (Comitato Etico Azienda Ospedaliero‐Universitaria Città della Salute e della Scienza, Torino) (n° protocol number: 0028226, 2021), and all participants provided written informed consent.

## Data Availability

The data supporting the findings of this study will be available upon request to the author.
